# Pyroptosis, a New Breakthrough in Cancer Treatment

**DOI:** 10.3389/fonc.2021.698811

**Published:** 2021-07-26

**Authors:** Dengqiang Wu, Changhong Wei, Yujie Li, Xuejia Yang, Sufang Zhou

**Affiliations:** ^1^ National Center for International Research of Bio-targeting Theranostics, Guangxi Key Laboratory of Bio-targeting Theranostics, Collaborative Innovation Center for Targeting Tumor Diagnosis and Therapy, Guangxi Talent Highland of Bio-targeting Theranostics, Guangxi Medical University, Nanning, China; ^2^ Department of Biochemistry and Molecular Biology, School of Pre-Clinical Science, Guangxi Medical University, Nanning, China

**Keywords:** pyroptosis, programmed cell death, gasdermin family, cancer treatment, antitumor immunity

## Abstract

The way of cell death can be roughly divided into two categories: cell necrosis and PCD(programmed cell death). Pyroptosis is a kind of PCD, its occurrence depends on the gasdermin protein family and it will produce inflammatory response. With constant research in recent years, more and more evidences show that pyroptosis is closely related to the occurrence and development of tumors. The treatment of tumors is a big problem worldwide. We focus on whether we can discover new potential tumor markers and new therapeutic targets from the mechanism. If we can understand the mechanism of pyroptosis and clear the relationship between pyroptosis and the development of tumors, this may provide a new reference for clinical cancer treatment.

## Background

Countless people die of tumors in the world every year (1), the prevention and treatment of tumors are very important to human health. The most conventional methods of cancer treatment are surgery, radiotherapy and chemotherapy. Pyroptosis is a type of PCD. Comparing with several other ways of death, pyroptosis occurs faster than others. And it is accompanied with a strong inflammatory response in the body. In recent years, more and more studies have shown that there was a close relationship between pyroptosis and tumors (2).

In 1986, Friedlander discovered that the use of anthrax lethal toxin could induce rapid lytic death of mouse macrophages, accompanied with non-specific leakage of intracellular materials (3). In 1992, Zychlinsky et al. found that it was morphologically different from the well-known apoptosis (4). At that time, they believed that it was a kind of PCD caused by the activation of caspase-1. In 1999, D. Hersh et al. showed that cell death caused by Salmonella Shigella could be blocked if caspase-1 was knocked out (5). In 2001, Cookson BT et al. first described this type of PCD accompanied with inflammation as pyroptosis (6), and distinguished this new form of cell death from others. Fink SL et al. defined pyroptosis in 2005 as caspase-1-mediated PCD that the cell underwent nuclear constriction, DNA fragmentation, swelling and finally rupture, accompanied with the release of inflammatory factors such as IL-1β (interleukin-1β) and IL-18 (interleukin-18) (7). GSDMD(gasdermin D) was identified as a key protein of pyroptosis in 2015, and study showed that caspase-1/4/5/11 could all cut the GSDMD protein (8, 9). One year later in 2016, further mechanism studies clarified that GSDMD-NT could oligomerize in biomembranes to form pores (10). Doctor Feng Shao reported that multiple gasdermin-N domain had the function of pore-forming and could induce pyroptosis in the same year (11). In 2017, Shi J et al. redefined the concept of pyroptosis as programmed cell necrosis mediated by gasdermin family proteins (12).

In 2018, NCCD (Nomenclature Committee on Cell Death) revised the definition of pyroptosis as a type of RCD (regulated cell death) that relies on the formation of cytoplasmic membrane pores by the gasdermin protein family, and its occurrence is often (but not always) as a consequence of inflammatory caspase activation (13) (**Table 1**).

**Table 1:** The discovery of pyroptosis.

Year	Authors	Discovery	Refs
In 1986	Friedlander	The lethal anthrax toxin induced rapid death of macrophages, accompanied with the leakage of intracellular materials.	(3)
In 1992	Zychlinsky et al.	It was different from the common apoptosis in morphology.	(4)
In 1999	D. Hersh et al.	Knocking out caspase-1, Salmonella Shigella could not cause cell death.	(5)
In 2001	Cookson BT et al.	It was the first time to describe this type of PCD accompanied with inflammatory response as pyroptosis.	(6)
In 2005	Fink SL et al.	Pyroptosis was defined as a kind of PCD mediated by Caspase-1.	(7)
In 2015	Shi et al.	GSDMD was identified as a key protein of pyroptosis and it was the common substrate of caspase-1/4/5/11.	(8, 9)
In 2016	Liu et al.	GSDMD-NT could oligomerize in biomembranes to form pores	(10, 11)
In 2017	Shi et al.	The concept of pyroptosis was redefined as PCD mediated by gasdermin family proteins.	(12)
In 2018	Galluzzi L et al.	The definition of pyroptosis was modified as a kind of RCD that depends on the gasdermin protein family to form pores in cytomembrane. Its occurrence was often(but not always) the result of inflammatory caspase activation.	(13)

## Molecular Mechanism of Pyroptosis

We have consulted the literatures on the mechanism of pyroptosis in recent years and made a certain summary.

Gasdermin was first reported in 2000 as a candidate gene for murine skin mutations. Since the protein was mainly expressed in the gastrointestinal tract and skin of mice, and its expression was restricted to the esophagus and stomach in the gastrointestinal tract, so it was named gasdermin (14). Gasdermin protein plays a decisive role in the process of pyroptosis, which is composed of two different domains, including GSDM-NT (N-terminal) and GSDM-CT (C-terminal) domains, and they are connected by a flexible connecting region. Without the cleavage by an activation signal, GSDM-CT can inhibit the activity of GSDM-NT by binding to it (11). Once GSDM-NT is released, it will form oligomers to play the function of drilling and induce pyroptosis (15).

## The Canonical Inflammasome-Induced Pyroptosis

**Figure 1:**
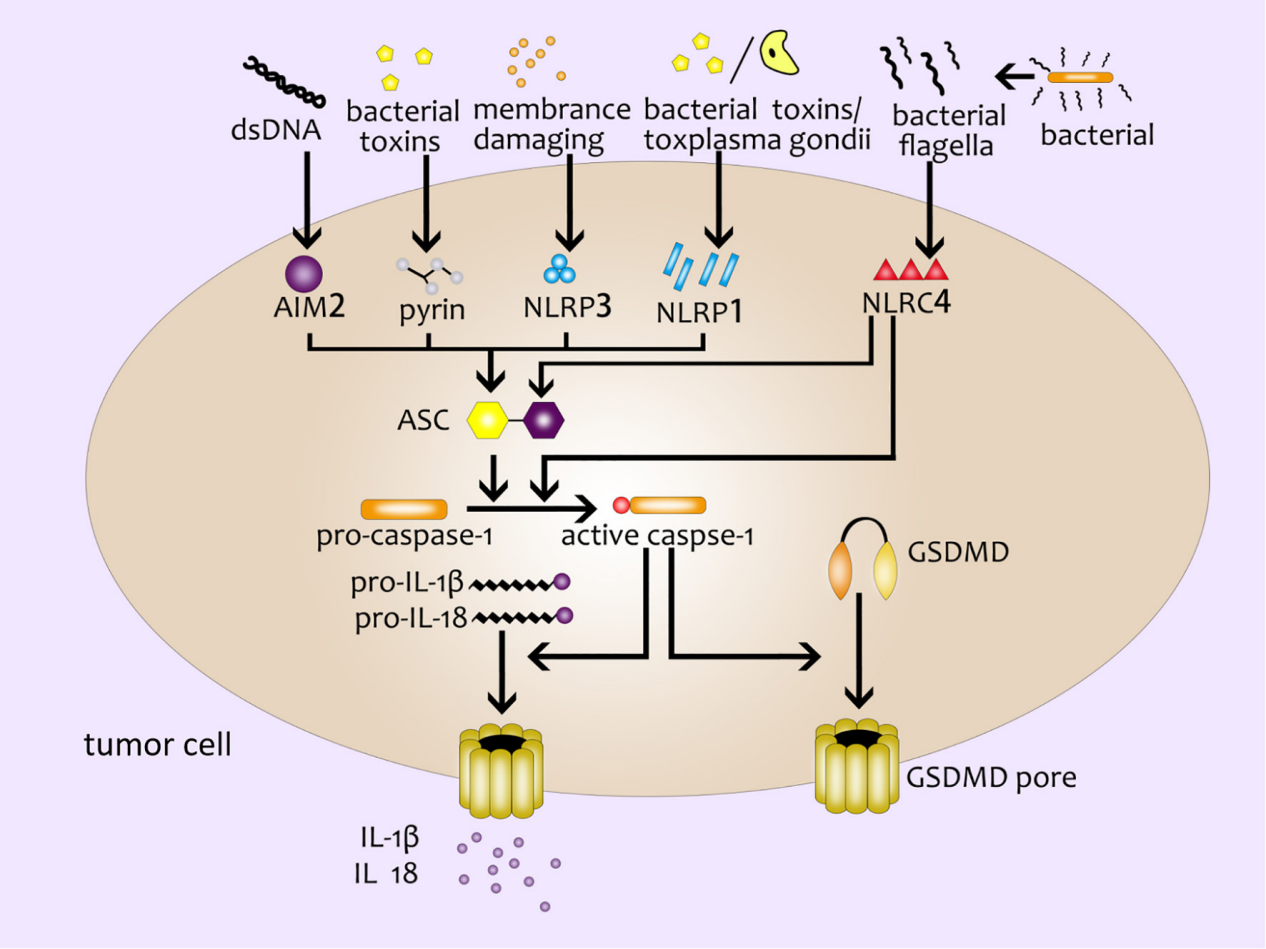
The canonical inflammasome pathway. Double-stranded DNA, bacterial toxins, membrane-damaging agents, toxoplasma gondii, bacterial flagella and other external stimulants can activate inflammasomes in cells and further recruit ASC and pro-caspase-1 to form inflammasome complex. Active caspase-1 cleaves GSDMD to produce GSDMD pores on the cell membrane; active caspase-1 can activate pro-IL-1β and pro-IL-18, and then IL-1β and IL- 18 are released from the GSDMD pores.

The pyroptosis mediated by the canonical pathway is mostly induced by the inflammasome complex (12). Several major inflammasomes have been discovered so far, including NLRP1 (NLR family pyrin domain containing 1) (16), NLRP3 (NLR family pyrin domain containing 3) (17), NLRC4 (NOD-like receptor containing a caspase activating and recruitment domain 4) (18), AIM2 (absent in melanoma2) (19–21) and the pyrin domain (22). These inflammasomes can sense the stimulation of various pathogenic signals, and interact with homotypic or heterotypic PYD/CARD (Caspase activation and recruitment domain). Meanwhile, they recruit the apoptosis-related dot-like protein ASC (adaptor protein apoptosis associated speck like proteins) and pro-caspase-1 to form the inflammasome complex (23, 24). Then caspase-1 is activated to cleave GSDMD to produce GSDMD-NT specifically (8, 25, 26). At the same time, Case CL et al. found that NLRC4 could directly bind to caspase-1 to cut GSDMD and induce pyroptosis without relying on ASC (27). Subsequently, active caspase-1 further cleave and activate pro-IL-1β and pro-IL-18 by recognizing the tetrapeptide sequence. IL-1βand IL- 18 are finally released outside the cell through the membrane pores (28) (**Figure 1**).

In addition to IL-1β and IL-18, IL-1α (29, 30) and HMGB1(high mobility group protein B1) (31) will also be released when pyroptosis, too.

## The Noncanonical Inflammasome-Induced Pyroptosis

**Figure 2:**
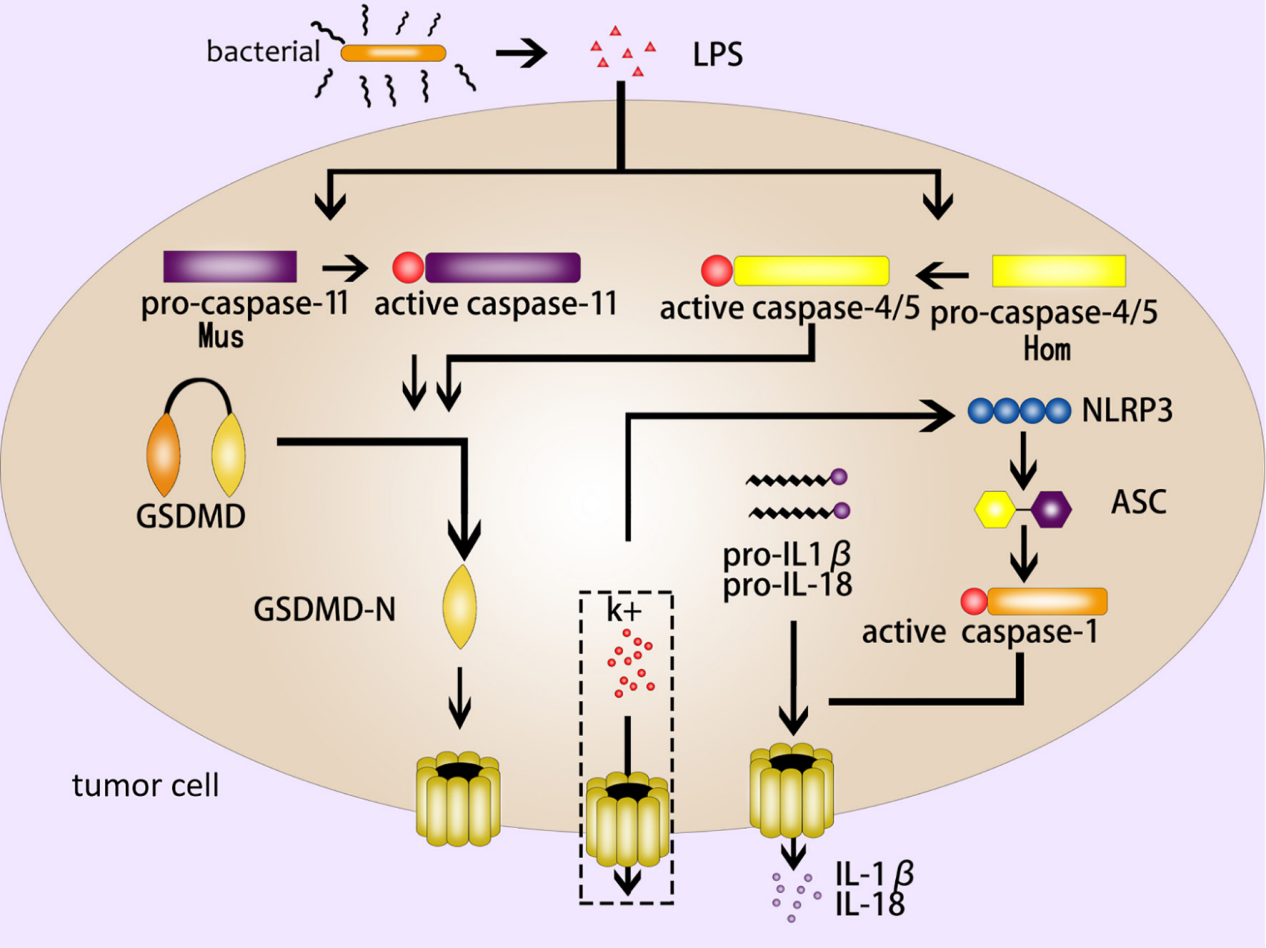
The noncanonical inflammasome pathway. The LPS of gram-negative bacteria activates caspase-11 (mouse) and caspase-4/5 (human), and the active caspase-11/4/5 cleave GSDMD to produce GSDMD-NT to form pores in the cell membrane; NLRP3 is activated by k+ efflux, which activates a series of pyroptosis-related proteins such as ASC and caspase-1, then caspase-1 induce GSDMD-mediated pyroptosis; IL-1β and IL- 18 are released from the GSDMD pores.

The noncanonical inflammasome pathway is different from the canonical inflammasome pathway due to its unique formation method. It mainly depends on the activation of caspase-11 (mouse) or caspase-4/5 (human) in the caspase family (2, 12). Different from caspase-1, the CARD domain of caspase-11 in mouse can directly recognize and bind to the LPS (lipopolysaccharide) of gram-negative bacteria, and present proteinase activity after being activated by oligomerization (32). GSDMD-NT cleaved by active caspase-11 form pores in cell membrane to induce pyroptosis (9, 33). On the other hand, the activation of caspase-11 by LPS can lead to the open of pannexin-1 (a nonselective macroporous protein channel) (34). The open of pannexin-1 can make a k+ efflux and activate NLRP3 inflammasome, inducing caspase-1-mediated pyroptosis (as mentioned above, the canonical inflammasome pathway) (35, 36). As for human caspase-4/5, LPS can stimulate them directly and then trigger the noncanonical inflammasome pathway (37) (**Figure 2**).

## Pyroptosis Mediated by Other Gasdermins

**Figure 3:**
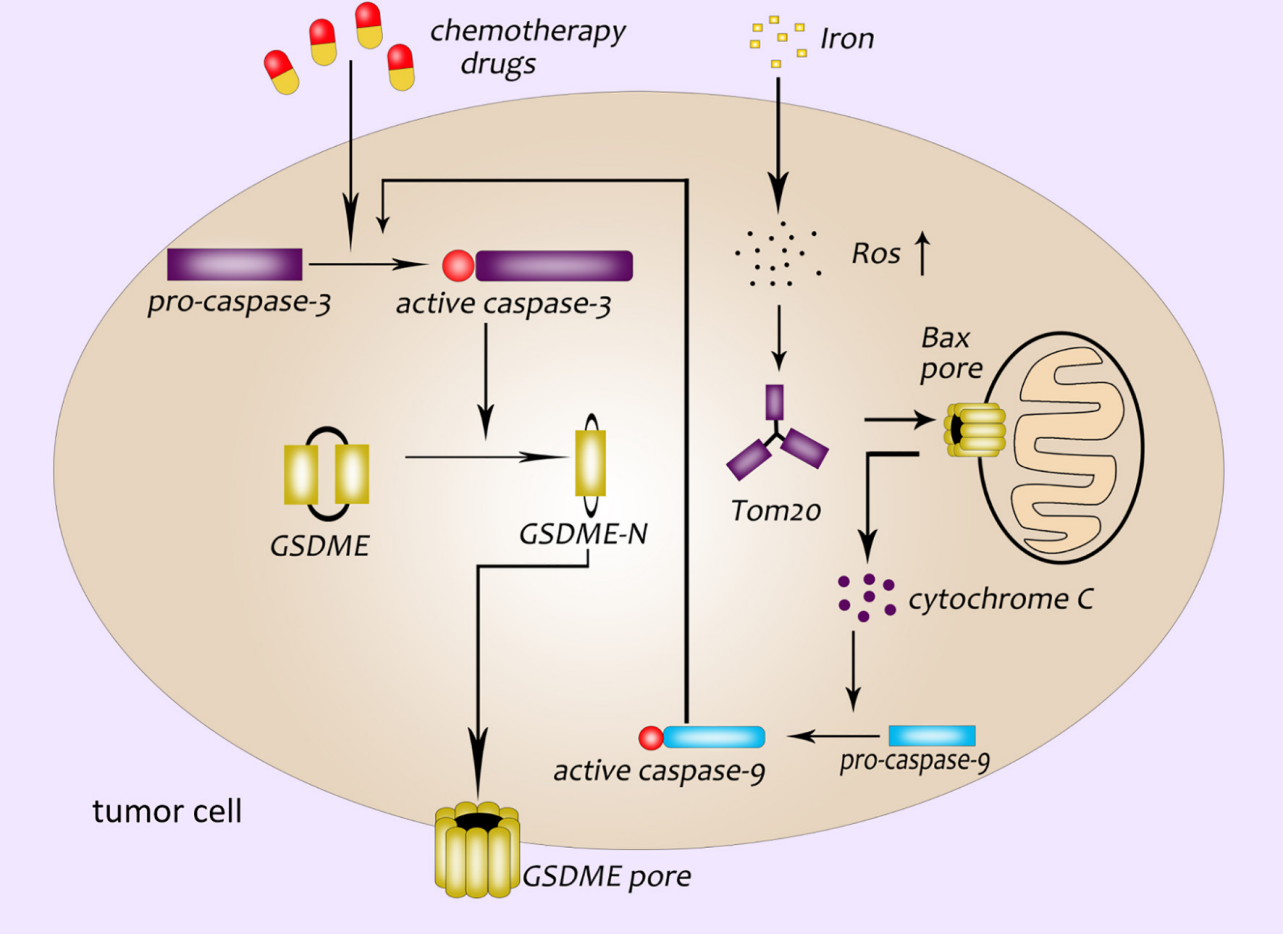
GSDME participates in the pathway of pyroptosis. Chemotherapeutics activate caspase-3; GSDME pore formation in the plasma membrane of cancer cells is dependent on the GSDME-NT cleaved by caspase-3; a newly discovered pyroptosis pathway related to ROS: ROS-Tom20-Bax-cytochrome C-caspase9-caspase3-GSDME.

GSDME was first identified as a deaf gene in 1988, also known as DFNA5 (deafness autosomal dominant 5) (38). Researches in recent years have found that GSDME was not only related to hearing damage, but also related to the occurrence of pyroptosis closely (39, 40). Both canonical and non-canonical inflammasome pathways are caused by GSDMD-NT. However, Wang et al. have demonstrated that cisplatin, paclitaxel and other conventional chemotherapeutic drugs can activate caspase-3. Then the active caspase-3 cleaves GSDME to form GSDME-NT, which leads to the appearance of pores in the cell membrane (40). Caspase-3 is originally an enzyme related to apoptosis (41–43). Wang et al. discovered that GSDME was a “switch” for chemotherapeutic drugs-induced apoptosis or pyroptosis in cancer cells. The cancer cells with high expression of GSDME undergo pyroptosis when treated with chemotherapeutic drugs while cells with low or no expression of GSDME undergo apoptosis (40). Excessive ROS (reactive oxygen species) in cells will enhance oxidative stress and cause cell necrosis. Wu et al. found that the iron-dependent intracellular ROS could be sensed by the outer mitochondrial membrane protein Tom20. And then the Bax protein translocate to the surface of the mitochondria to form a cavity, which leads to the release of cytochrome C. Cytochrome C activates caspase-9, and caspase-9 further activates caspase-3, active caspase-3 induces GSDME-mediated pyroptosis (44) (**Figure 3**). In addition, it was found that Granzyme B (GZMB) could cleave GSDME like caspase-3 and induce pyroptosis, too (45).

**Figure 4:**
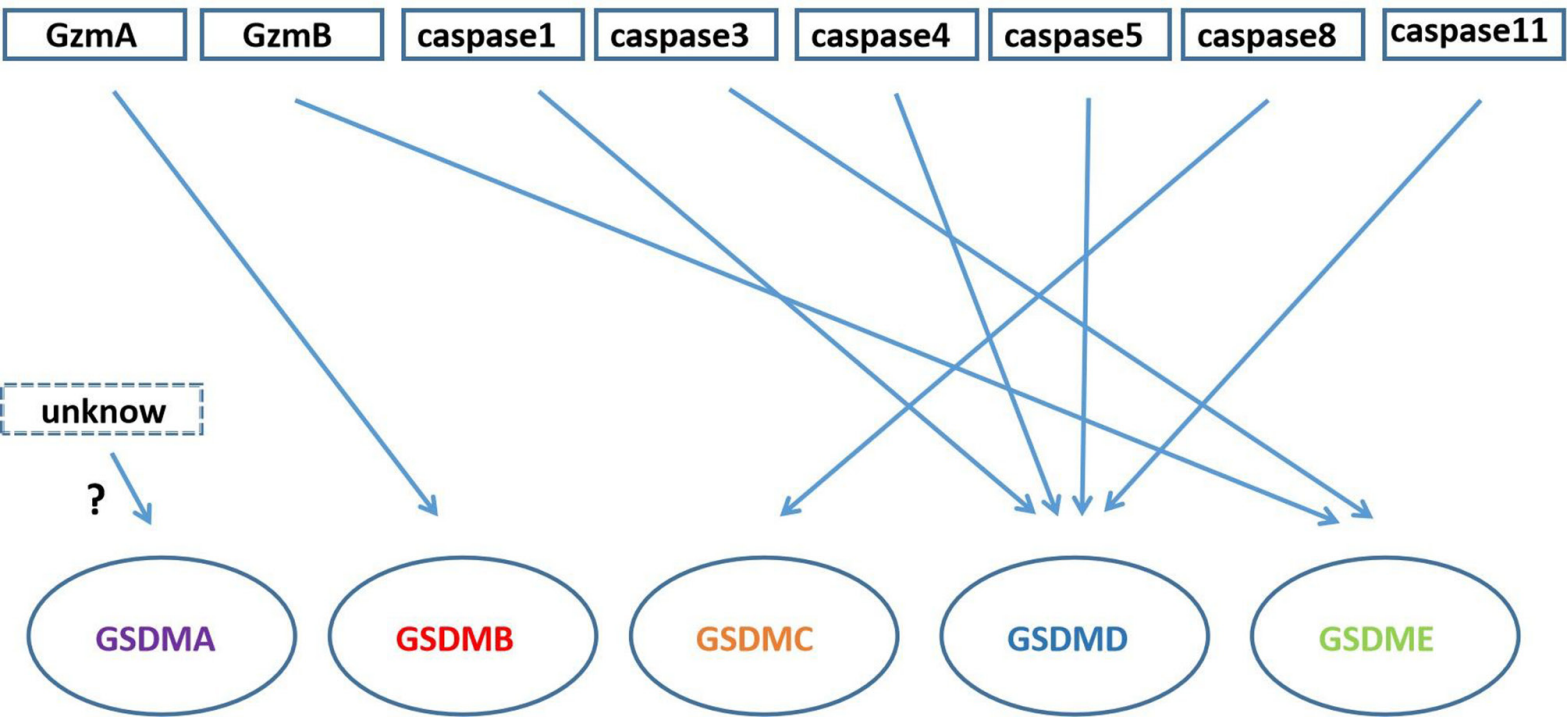
Gasdermin proteins cleaved by Granzyme and caspase family. GzmA cleaves GSDMB; caspase3/GzmB cleaves GSDME; caspase-1/4/5/11 cleave GSDMD; caspase-8 cleaves GSDMC; there is unknow what can cleave GSDMA.

In addition to GSDMD and GSDME proteins, other members of the gasdermin family such as gasdermin A, gasdermin B and gasdermin C also play an important role in pore formation and pyroptosis (**Figure 4**).

GSDMA has only one transcriptional copy in the human genome, while mice have three (Gsdma1-3) (46). GSDMA-NT and Gsdma3-NT have similar effects to GSDMD-NT and GSDME-NT, both of which can bind to cell membrane lipids (cardiolipin or phosphoinositide), and then induce pore formation (11). But how GSDMA is cleaved has not been reported in the literatures yet.

GSDMB has the same site (17q21.1) as GSDMA in human chromosomes (14, 47), while the GSDMB gene is not detected in mouse. GSDMB is specifically expressed in the epithelium of the esophagus and gastrointestinal tract, the bronchial epithelium of the asthmatic lung (48). In addition, GSDMB is expressed in liver, neuroendocrine and immune cells, too (49, 50). GSDMB-NT domain can induce pyroptosis in HEK293T cells (11), and Panganiban et al. found that caspase-1 can cleave full-length GSDMB at site 236 (51). However, the study by Chao et al. mentions that GSDMB is not a substrate of inflammatory caspases-1/4/5/11 because it lacks specific interdomain junction regions. At the same time, they prove that GSDMB is the substrate of caspase-3/6/7 and mean that the caspase recognition site is the n-terminal domain of GSDMB, not the interdomain junction region (52). Therefore, the complete GSDMB-NT domain cannot be generated by cutting with caspase-3/6/7, and it is not clear whether this cleavage product can induce pyroptosis. Recent studies have found that granzyme A could cleave GSDMB at Lys244, and the GSDMB-NT have pore-forming activity (53). All in all, people are divided on the role of GSDMB in pyroptosis and the mechanism of caspase with GSDMB needs further research.

GSDMC has also been confirmed to play a role in pyroptosis. GSDMC was initially found to be highly expressed in metastatic melanoma cells, and was called MLZE (melanoma-derived leucine zipper-containing extranuclear factor) (54). In the study of Hou et al. it was found that caspase8 could cleave GSDMC and GSDMC could switch TNF-α-induced apoptosis to pyroptosis in MDA-MB-231 breast cancer cells under the death receptor signaling (55).

## The Differences and Similarities Between Pyroptosis, Apoptosis, Necroptosis and Autophagy

We make a table to distinguish them from the definition, basic characteristics and some check indexes (**Table 2**).

**Table 2:** The difference and similarities between the four types of death.

	Pyroptosis	Apoptosis	Necroptosis	Autophagy	Refs
Definition	A regulable cell death that relies on the GSDM protein family to form pores in the membrane of the cell. Its occurrence is often the result of inflammatory Caspase activation.	One is characterized by the activation of cysteine proteases (caspase). The initiator caspase receives external or internal apoptotic signals, and then activates the executor caspases to initiate the death program.	A form of RCD(regulated cell death), induced by disturbances of extracellular or intracellular homeostasis, is heavily dependent on the MLKL, RIPK1(in some times), and RIPK3.	A form of RCD (regulated cell death) that mechanistically depends on the autophagic machinery (or components thereof).	(13)
Inflammation	Yes	no	Yes	no	(23, 57–59)
Morphological characteristic	Swell	Shrink	Swell	Crescent-shaped or cup-shaped	(60, 61)
Cell membrane	pore formation	Intact	pore formation	Intact	(58, 62, 63)
Organelle	Intact/deformed	intact	Swell	engulfed by autophagosomes	(56, 58, 62)
Pore-forming protein	gasdermin protein	no	MLKL	no	(11, 63–65)
IL-18, IL-1β release	Yes	no	Yes	no	(11, 28, 62)
IL-1α release	Yes	no	Yes	no	(29, 30, 62)
HMGB1 release	Yes	no	Yes	no	(31, 66, 67)
LDH release	Yes	no	Yes	no	(8, 9)
DNA degradation	Random degradation	Ladder degradation	Random degradation	Random degradation	(60, 64)
Annexin V/PI staining	+/+	+/-	+/+	+/-	(68, 69)

## Pyroptosis With Tumors

### Lung Cancer and Pyroptosis

Lung cancer is one of the most harmful malignant tumors in the world. In China, lung cancer deaths account for more than 1/5 of all tumor deaths (70). TP53 was originally a tumor suppressor gene (71–73), and Tianze Zhang et al. found that it had some connections with pyroptosis. In NSCLC(nonsmall-cell lung cancer) tissues, p53 is positively correlated with pyroptosis. In A549 cells, overexpression and silence of p53 can correspondingly regulate the occurrence of pyroptosis, and p53 can induce pyroptosis (74). Gao et al. found that the expression of GSDMD in lung cancer tissues of patients with NSCLC was significantly higher than that in adjacent tissues.

MTT and colony experiments proves that the GSDMD-knockout can inhibit the growth of PC9, H1703 and H1975 cell lines of NSCLC (75). Cisplatin and paclitaxel are common chemotherapeutics used to treat tumors (76–78). In the study of Zhang et al., it was found that both cisplatin and paclitaxel could induce apoptosis and pyroptosis of A549 cells, but the effect of cisplatin was strong than taxol (79). The chemotherapeutic drug cisplatin regulates the release of immune factors in the induction of GSDME-mediated pyroptosis, and the level of MIP-1α, MIP-1β, MIP-2 and IP-10 in tumor tissues and blood are increase. Peng et al. think that it is precisely the release of immune factors that recruits T cells in tumor tissues and trigger anti-tumor effect (80).

TTM (Trillium tschonoskii Maxim) is a traditional Chinese medicine has anti-tumor effects (81, 82). Recent studies have found that PPVI (Polyphyllin VI) extracted from TTM could activate caspase-1 through the ROS/NF-κB/NLRP3/GSDMD signaling pathway and turn apoptosis to pyroptosis in NSCLC cells (83). Huaier is also a traditional Chinese medicine have the anti-tumor effects (84). Huaier extract can induce NLRP3- mediated pyroptosis and inhibit the development of NSCLC, which provides a new reference idea for clinical treatment about NSCLC (85).

### Osteosarcoma and Pyroptosis

Osteosarcoma is a malignant tumor that originates from mesenchymal tissues. It is characterized by malignant spindle-shaped stromal cells that can produce bone-like tissue. It is a common primary malignant bone tumor in adolescents (86). The current conventional treatment method is radical surgical resection combined with neoadjuvant chemotherapy (87). Due to the combination of surgical resection and chemotherapy, the 5-year survival rate has been significantly improved (88). However, because of the side effects of chemotherapy drugs, it is still necessary to find new drugs. PG (Emodin 80O-β-glucopyranoside) is one of the main active components of R. Japonicas, and more and more studies have reported that PG had an important influence on the progression of various malignant tumors (89, 90). Osteosarcoma cells HOS and SAOS-2 undergo pyroptosis after using PG. At the same time, the anti-tumor effect of PG on HOS and SAOS-2 cells is mediated by the NLRP3 inflammasome activated by endoplasmic reticulum stress. In addition, *in vivo* experiments showed that PG reduced the growth and invasion of tumor in osteosarcoma mouse models. These results suggest the possible mechanism of the effect about PG in human osteosarcoma cells and provide a new drug choice for the clinical treatment of osteosarcoma (91).

Dioscin is a kind of steroidal saponins extracted from medicinal plants, including polygonatum, dioscorea nigra and dioscorea zingiberensis (92–94). Recent studies have reported that it also had an inhibitory effect on tumors. Osteosarcoma cells can produce GSDME-NT after being treated with dioscin. Meanwhile, the effect of dioscin in osteosarcoma cells is significantly reduced when GSDME knocked out by the specific siRNA (Small interfering RNA). This study also found that dioscin induced G2/M phase arrest and apoptosis through the JNK/p38 pathway to inhibit the growth of osteosarcoma cells (95). Pyroptosis can trigger cell apoptosis under certain conditions so that the two can work together to exert their anti-osteosarcoma function.

B.-G. TIAN et al. found that miRNA-181a was abnormally elevated in osteosarcoma tissues and cells. In addition, down-regulation of miRNA-181a can activate NLRP3-mediated pyroptosis (96). Compared with normal tissues, the expression of GSDMD in osteosarcoma is relatively high, which can independently indicate the prognosis status of patients with osteosarcoma. Rongjin Lin et al. also believe that GSDMD protein may play an important role in the progression and resistance of osteosarcoma (97).

### Breast Cancer and Pyroptosis

Breast cancer is one of the most common malignant tumors that endanger women’s health in the world (98, 99). Compared with other types of tumors, the incidence of breast cancer is extremely high and shows a trend of increasing and the patients are younger and younger year by year (98, 100). In 108 cases of breast cancer and 23 cases of para-carcinoma tissues, Wu et al. detected the expression level of caspase-1, IL-1βand GSDMD, and they found that the expression of pyroptosis-related proteins were inversely correlated with the tumor grade, tumor size, clinical stage, death risk of breast cancer tissues (101). This also means that proteins like caspase-1, IL-1β and GSDMD may affect the development and prognosis of breast cancer, providing new molecular targets for the clinically targeted treatment of breast cancer.

DHA (docosahexaenoic acid) is an omega-3 fatty acid with anti-cancer effect. It can inhibit the growth of breast cancer cells, increase apoptosis, and reduce cell invasiveness (102, 103). However, Nathalia et al. added DHA to breast cancer cells MDA-MB-231 and found that the activity of caspase-1 and GSDMD were enhanced, the secretion of IL-1β was increased, and showed pore-formation activity, which suggesting the occurrence of pyroptosis (104). This discovery provides a new idea for the anti-cancer effect of DHA.

PD-L1 (programmed death ligand 1) on the surface of cancer cells can inhibit anti-tumor immunity by interacting with its receptor PD-1 (programmed cell death protein 1) (105, 106). Therefore, blocking the PD-L1/PD-1 interaction can enhance the body’s anti-tumor immunity (105). This is a new major breakthrough in cancer treatment (107, 108). Studies have reported that PD-L1 was localized on the nuclear (nPD-L1) in breast cancer cells treated with doxorubicin (109, 110), but the function and mechanism of nPD-L1 were not yet clear. The study of Hou et al. have showed that under hypoxic condition, PD-L1 could translocate to the nuclear (nPD-L1) through p-Y705-Stat3. What’s more, nPD-L1 could switch TNFα-induced apoptosis to pyroptosis and GSDMC was cleaved by caspase8 (55). The functionality of a non-immune checkpoint of PD-L1 is clarified here, which is a completely new understanding of PD-L1. At the same time, they also found that some chemotherapeutics such as daunorubicin, doxorubicin, epirubicin and actinomycin D could induce the expression of nPD-L1 (nuclear PD-L1) and GSDMC as well as the activation of caspase-8, inducing pyroptosis in MDA-MB-231 human breast cancer cells (55).

### Hepatocellular Carcinoma and Pyroptosis

HCC (hepatocellular carcinoma) has the sixth highest incidence rate in the global cancer incidence rate and the second highest mortality rate in the global cancer mortality rate (111). HCC patients in China account for about 55% of new HCC cases worldwide each year (112). The main clinical treatments for liver cancer include chemotherapy, radiotherapy, surgical resection and interventional therapy, but chemotherapy and radiotherapy have certain side effects (113).

Recently, it has been reported that the induction of pyroptosis of HCC cells can inhibit the growth of liver cancer (114). Wei et al. found that the expression of NLRP3 and ASC, the components of the NLRP3 inflammasome, were significantly lower than that of paracancer tissues. The expression of NLRP3 was significantly down-regulated when HCC. With the development of the pathological grade and clinical stage of HCC patients, the expression of inflammatory components of NLRP3 are decreased (115). Later, they found that the E2 (17β-estradiol)-induced death of HCC cells was related to caspase1-mediated pyroptosis, and proved that NLRP3 inflammasome inhibited autophagy through the E2/ERβ/AMPK/mTOR pathway (116). E2 induces pyroptosis and inhibits autophagy, which significantly slows down the development of HCC.

AIF (Alpinumisoflavone) is the principal bioactive component of derriseriocarpa sourced from China that has the effects of anti-tumor (117, 118). For liver cancer, Zhang et al. found that AIF could also inhibit the growth and metastasis of HCC cells by inducing NLRP3-mediated pyroptosis. *In vivo* experiments, they found that AIF could inhibit tumor growth and increase the expression of pyroptosis-related proteins in tumor tissues (114). Euxanthone is a flavonoid isolated from the Polygala Caudata plant. It was used as a traditional Chinese medicine to treat coughs in ancient China (119). Recent studies have shown that it has anti-tumor effects (120, 121). Chen et al. collected samples from hospital patients and found that the expression of caspase-1, IL-1β and IL-18 were low in both HCC tissues and HCC cell lines. At the same time, they found that Euxanthone could inhibit the development of HCC by inducing pyroptosis (122).

### Gastric Cancer and Pyroptosis

Gastric cancer is one of the common malignant tumors (123). Among the malignant tumors surveyed in 2018, the incidence and mortality of gastric cancer ranked fifth and second. Early gastric cancer is difficult to detect, 80% of patients are in the middle and advanced stages when they see a doctor, and the 5-year survival rate is only about 30% (124). Chemotherapy drugs are often used to treat the gastric cancer, but the mechanism is still unclear. Studies have found that there is a certain relationship between gastric cancer and pyroptosis (125).

Wang et al. found that gastric cancer cell lines SGC-7901 and MKN-45 underwent pyroptosis when treated with 5-FU(5-fluorouracil), and they proved that caspase-3 was activated by 5-FU to induce GSDME-mediated pyroptosis (126). The same gastric cancer cell line SGC-7901 showed obvious pyroptosis characteristics after using BIX-01294 combined with cisplatin. And GSDME-knockout could convert the pyroptosis into apoptosis under the same condition (127).

In gastric cancer tissues, the expression of GSDMD was lower than normal. Studies have found that downregulating the expression of GSDMD could promote the development of gastric cancer. At the meanwhile, Wang et al. speculated that the downregulation of GSDMD may regulate cell cycle-related proteins by activating ERK (extraCellular signal-regulated kinase), STAT3 (Signal Transducer and Activator of Transcription 3) and PI3K/AKT (phosphatidylinositol 3 kinase) signaling pathway, and promoted the S/G2 transition of gastric cancer cells. Therefore, GSDMD has certain clinical significance in the targeted therapy and diagnosis of gastric cancer (128).

### Other Types of Cancer and Pyroptosis

Nobiletin is a food phytochemical extracted from citrus fruits (129). It has been reported that it could inhibit the growth of ovarian cancer (130, 131). Zhang et al. found that nobiletin could reduce the mitochondrial membrane potential, induce ROS generation, and play a role in GSDMD/GSDME-mediated pyroptosis in HOCC (human ovarian cancer cells). In summary, nobiletin may become a new anti-ovarian cancer drug, which can trigger apoptosis and induce pyroptosis by regulating the autophagy of ovarian cancer cells (132).

ESCC (esophageal squamous cell carcinoma) is a kind of gastrointestinal cancer. Due to the resistance of cancer cells to chemotherapy drugs such as cisplatin and 5-fluorouracil, therapeutic effects are often unsatisfactory (133). Metformin is a kind of anti-diabetic drug (134). A study reported that metformin could induce GSDMD-mediated pyroptosis of ESCC by targeting the miR-497/PELP1 axis (135).

In addition, Yu et al. found that the expression of miR-214 and NLRP3 were down-regulated in cervical cancer patients, while the up-regulation of miR-214 could promote the pyroptosis of cervical cancer cells by targeting the expression of NLRP3 (136).

Endometrial cancer is one of the most common cancers in gynecology (137). Studies have reported that pyroptosis-related protein caspase-1, NLRP3 and GSDMD were highly expressed in endometrial cancer tissue, and hydrogen could inhibit the growth of endometrial cancer by inducing GSDMD-mediated pyroptosis through a ROS/NLRP3/caspase-1 pathway (138).

PDAC(Pancreatic ductal adenocarcinoma) accounts for 95% of pancreatic malignancies. Despite decades of effects, its five-year survival rate is still only about 8% and the incidence is increasing year by year (139). The expression of MST1 (STE20-like kinase 1) in PDAC is decreased. Restoring the expression of MST1 can promote PDAC cell death, and inhibit the proliferation, migration and invasion of PDAC cells by inducing caspase-1-mediated pyroptosis *via* ROS (140).

Because colitis is one of the risk factors in colorectal cancer, CAC (colitis-associated colorectal cancer) accounts for about 5% of colorectal cancer cases (141). CAC is one of the examples of chronic inflammation related cancers and chronic inflammation is present in the early stage of tumor onset (142). It has been found that GSDME-mediated pyroptosis induced cancer cells proliferation and PCNA(proliferating cell nuclear antigen) expression through the ERK1/2 pathway by releasing intracellular HMGB1, which in turn promoted the development of CAC (143). The results of the study emphasize the new role of HMGB1 in promoting the tumorigenesis of CAC, so it may become a new strategy to inhibit GSDME-mediated pyroptosis or use neutralizing anti-HMGB1 antibodies in the treatment of CAC.

The small molecule inhibitors of serine dipeptidase DPP8 and DPP9 can induce pyroptosis in mouse and human monocytes/macrophages (144, 145). Johnson et al. found that CARD8 (caspase activation and recruitment domain) can regulate pro-caspase-1-mediated-pyroptosis in human myeloid cells induced by the inhibitor DPP8/9. Further studies have shown that DPP8/9 inhibitors-induced pyroptosis in most human AML (acute myeloid leukemia) cell lines and primary AML samples, and these inhibitors also have effects on inhibiting human AML in mouse models (146) (**Table 3**).

**Table 3:** Tumors and pyroptosis.

Tumor types	Discovery	Refs
Lung cancer	1. p53 can induce pyroptosis (Gao et al.); 2. chemotherapeutic drug cisplatin can indue GSDME-mediated pyroptosis (Peng et al.); 3. PPVI can active caspase-1 and turn apoptosis to pyroptosis of NSCLC cells through the ROS/NF-κB/NLRP3/GSDMD pathway (Teng JF et al.); 4. Huaier extract can activate NLRP3 to induce pyroptosis and inhibit the development of NSCLC (Xie Jet al.).	(74, 75, 80, 83, 85)
Osteosarcoma	1. PG (Emodin 80O-β-glucopyranoside) can reduce tumor growth and invasion in osteosarcoma mouse models by inducing pyroptosis (Tian B et al.); 2. Dioscin can induce GSDME-mediated pyroptosis in osteosarcoma cells (Ding Q et al.); 3. MiRNA-181a is abnormally elevated in osteosarcoma tissues and cells and the down-regulation of miRNA-181a can induce NLRP3-mediated pyroptosis (B.-G. TIAN et al.); 4. The expression of GSDMD is relatively high in osteosarcoma tissues compared to normal (Rongjin Lin et al.).	(91, 95–97)
Breast cancer	1. Pyroptosis-related protein such as caspase-1, IL-1β, and Gasdermin-D are negatively correlated with the tumor grade, tumor size, clinical stage, death risk of breast cancer (Wu et al.); 2. DHA (docosahexaenoic acid) can induce GSDMD-mediated pyroptosis of breast cancer cells (Nathalia et al.); 3. Under hypoxic condition, nPD-L1 can switch TNFα-induced apoptosis to pyroptosis and GSDMC is cleaved by caspase8 in breast cancer cells (Hou et al.).	(55, 101, 104)
Hepatocellular carcinoma	1. The expression of NLRP3 and ASC in HCC tissues were significantly lower than paracancer tissues (Wei et al.); 2. NLRP3 inflammasomes induce pyroptosis and inhibit autophagy through the E2/ERβ/AMPK/mTOR pathway (Wei et al.); 3. AIF can inhibit the growth of HCC both *in vitro* and *in vivo* by inducing NLRP3-mediated pyroptosis (Zhang et al.); 4. Euxanthone can inhibit the development of HCC by inducing pyroptosis (Chen et al.).	(114–116, 122)
Gastric cancer	1. Caspase-3 was activated by 5-FU to induce GSDME-mediated pyroptosis in gastric cancer cell lines SGC-7901 and MKN-45 (Wang et al.); 2. Knocking out GSDME in SGC-7901 cells can convert the pyroptosis into apoptosis after using BIX-01294 combined with cisplatin (Deng BB et al.); 3. The expression of GSDMD in gastric cancer is lower than normal tissues (Wang et al.); 4. A speculation that the downregulation of GSDMD may regulate cell cycle-related proteins by activating ERK, STAT3 and PI3K/AKT pathway, and promote the S/G2 transition of gastric cancer cells (Wang et al.).	(126–128)
Ovarian cancer	Nobiletin can trigger apoptosis and induce GSDMD/GSDME-mediated pyroptosis by regulating the autophagy of ovarian cancer cells (Zhang et al.).	(132)
ESCC (Esophageal squamous cell carcinoma)	Metformin can induce GSDMD-mediated pyroptosis of ESCC by targeting the miR-497/PELP1 axis (Lu Wang et al.).	(135)
Cervical cancer	The up-regulation of miR-214 can promote the pyroptosis of cervical cancer cells by targeting the expression of NLRP3 (Yu et al.).	(136)
Endometrial cancer	Hydrogen can inhibit the development of endometrial cancer by induce GSDMD-mediated pyroptosis through a ROS/NLRP3/caspase-1 pathway (Ye Yang et al.).	(138)
PDAC (Pancreatic ductal adenocarcinoma)	The expression of MST1 in PDAC is decreased and overexpression of MST1 can promote PDAC cell death by inducing caspase-1-mediated pyroptosis *via* ROS (Cui et al.).	(140)
CAC (colitis-associated colorectal cancer)	GSDME-mediated pyroptosis can induce cancer cells proliferation and PCNA expression through the ERK1/2 pathway by releasing intracellular HMGB1 (Tan et al.).	(143)
AML (acute myeloid leukemia)	DPP8/9 inhibitors-induced pyroptosis in most human AML cell lines and primary AML samples, and these inhibitors also have an effect on inhibiting human AML in mouse models (Johnson et al.).	(146)

## Antitumor Immunity

Pyroptosis in tumor cells can induce antitumor immunity (147–150). In GSDME-expressing tumor cells, the damage-associated molecular patterns (DAMPs) generated by the pyroptosis of the cells can recruit immune cells to the tumor microenvironment and enhance their immunity. The expression of GSDME greatly increases the number of TILs (tumor infiltrating lymphocytes) and the phagocytic capacity of macrophages (151).

Granzyme B has the same cleavage site as caspase-3 in NK cells, which can cleave GSDME to induce pyroptosis (45). Due to the occurrence of pyroptosis, it further promotes antitumor immunity and inhibits tumor growth. In immunodeficient mice and mice lacking NK cells and CD8+ killer T cells, the inhibitory effect of GSDME on tumors disappeared, indicating that this inhibitory effect depends on these two killer cells in the immune system (45). Cytotoxic lymphocytes [such as CTL (Cytotoxic T Lymphocyte) cells and NK cells] are important effector cells of the immune system. They release perforin and other mediators to play a killing role after recognizing target cells (152, 153). At present, the killing effect of lymphocytes is still generally considered to trigger apoptosis on target cells (154). However, Zhou et al. found that Granzyme A could enter tumor cells *via* perforin and induce GSDMB-mediated pyroptosis. Then up-regulating the expression of GSDMB can promote pyroptosis (53).

GSDMB is highly expressed in tumor cells derived from epithelial cells of the digestive system and GSDMB-mediated pyroptosis can enhance the antitumor immunity. This phenomenon also explains the killing mechanism of cytotoxic lymphocytes from some perspectives. In addition, Wang et al. used the bioorthogonal system to reveal that a small number of tumor cells undergo pyroptosis, which is sufficient to effectively regulate the tumor immune microenvironment, thereby activating a strong T cell-mediated antitumor immune response (155). This discovery provides a new idea for the research and development of tumor immunotherapy drugs. Gasdermin family proteins also have become potential biomarkers for tumor immunotherapy. The stimulus of gasdermin proteins is likely to become a new direction for the development of antitumor drugs.

**Figure 5:**
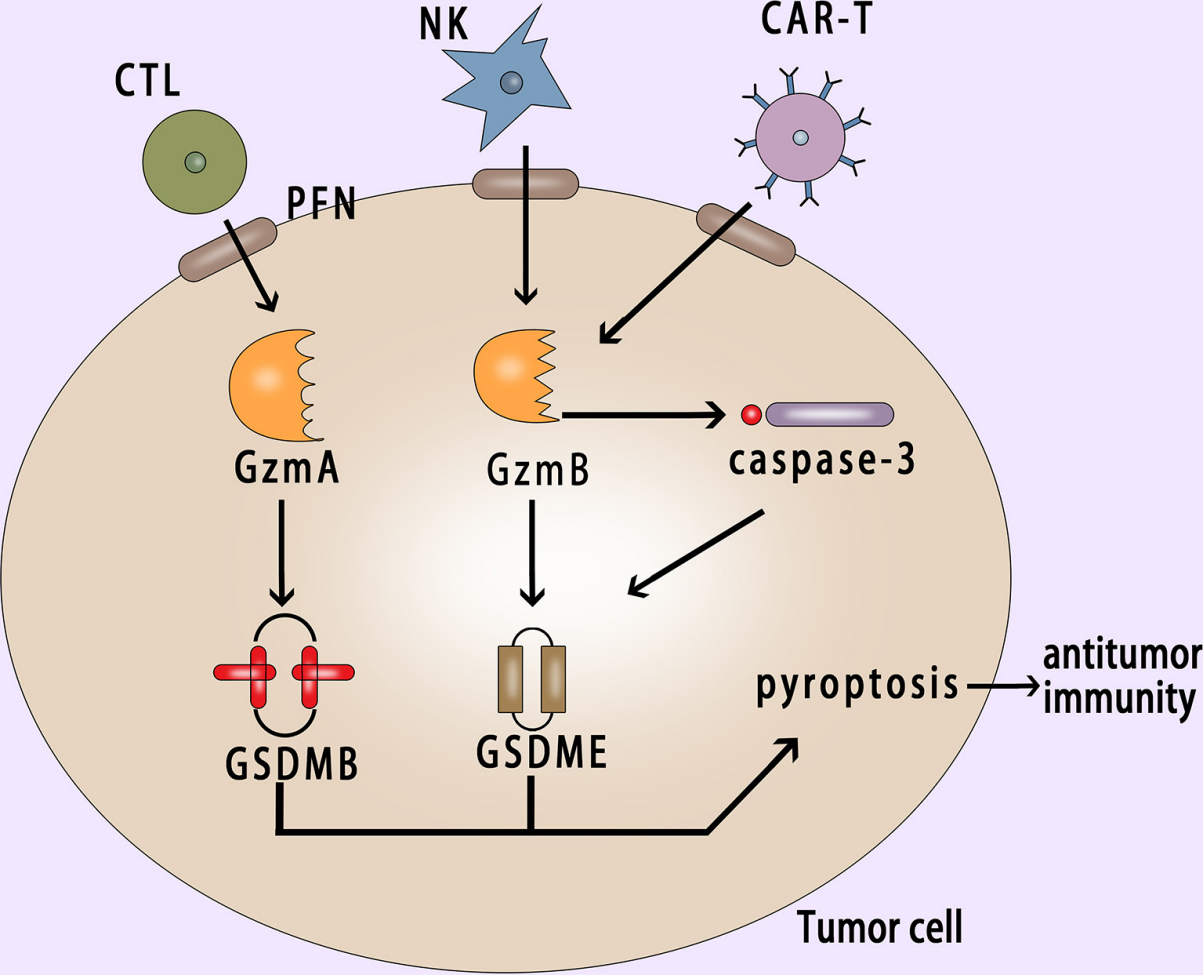
Pyroptosis in cancer cell activates antitumor immunity. GzmA and GzmB released by CTL and NK cells with the help of perforin enter into the cancer cell and induce the GSDMB-mediated and GSDME-mediated pyroptosis respectively; CAR-T cells release granzyme B, which can activate caspase-3 or cleave GSDME directly to induce pyroptosis; Pyroptosis can suppress tumor growth and promote antitumor immunity. PFN, perforin; NK, natural killer; CAR-T, chimeric antigen receptor T cell.

CAR-T (chimeric antigen receptor T cell) immunotherapy is a new type of targeted therapy for the treatment of hematologic malignancies (156–159). In recent years, good results have been achieved in the CAR-T immunotherapy. But CRS (cytokine release syndrome) is an important side effect of CAR-T therapy (160–162), and the mechanism of CRS is not yet clear. Liu et al. found that CAR-T cells released granzyme B, which activated caspase-3 and then cleaved GSDME or cleaved GSDME directly to cause pyroptosis (163) (**Figure 5**). Studies have showed that GSDME-knockout in tumor cells could eliminate CRS, which provided a new reference for reducing CRS after using CAR-T therapy clinically.

## Conclusion

Pyroptosis is a new type of PCD. Its induction depends on the gasdermin protein family and is often activated by caspase (164). GSDMD and GSDME of the gasdermin protein family are the most common types in the research. GSDMD is related to the canonical and noncanonical pathway of pyroptosis. In addition, the gasdermin protein family includes GSDMA, GSDMB, GSDMC and Pejvakin, too. Except for the Pejvakin protein, the others all have the function of pore-formation (165, 166).

Recent researches have found that pyroptosis was related to many types of diseases, such as cardiovascular diseases, metabolic diseases, immune-related diseases and cancers (167–170). Cancer is very harmful to human health and the current treatments for cancer are limited. Apoptosis-induction in tumor cells is a common way in clinical treatment of tumors (43). Due to the anti-apoptotic effect of tumor cells, pyroptosis, a new cell death way, has great potential in the treatment of tumors. There are many reports showed that some drugs can induce pyroptosis in tumor cells and these drugs can inhibit tumor growth *in vivo* experiments, too. The expression of pyroptosis-related proteins such as gasdermin family, caspase family, NLRP3, ASC, etc. are mostly different between tumor tissues and normal tissues. These protein molecules may become new targets for tumor progression and treatment.

It is a recent discovery that pyroptosis can induce antitumor immunity (45, 149). If we can utilize tumor pyroptosis to stimulate a stronger immune function in the body, this may become another major advancement in tumor treatment. At present, people’s understanding about pyroptosis is still superficial. And the mechanism of pyroptosis is still needs us to explore, more experiments and clinical trials are needed to explore its actual value and clinical application.

## Author Contributions

DW and CW drafted the manuscript. DW, YL, XY, and SZ discussed and revised the manuscript. SZ designed the research. All authors contributed to the article and approved the submitted version.

## Funding

This work was funded by the Guangxi Science and Technology Research Base and Talent-specific Project (AD18126021); National Science and Technology Major Project for New Drug Innovation (2018ZX09733001-004-002); National Natural Science Foundation of China (NSFC) project (No. 81872491); Key Laboratory of the Ministry of Education Project for Early Prevention and Treatment of Regional High-risk Tumors (GKE2018-03, GKE2019-09, GKE-ZZ202007).

## Conflict of Interest

The authors declare that the research was conducted in the absence of any commercial or financial relationships that could be construed as a potential conflict of interest.

## Publisher’s Note

All claims expressed in this article are solely those of the authors and do not necessarily represent those of their affiliated organizations, or those of the publisher, the editors and the reviewers. Any product that may be evaluated in this article, or claim that may be made by its manufacturer, is not guaranteed or endorsed by the publisher.

## Abbreviations

PCD, programmed cell death; IL-1β, interleukin-1β; IL-18, interleukin-18; GSDMD, gasdermin D; GSDM NT, gasdermin N-terminal; GSDM CT, gasdermin C-terminal; NLRP1, NLR family pyrin domain containing 1; NLRP3, NLR family pyrin domain containing 3; NLRC4, NOD-like receptor containing a caspase activating and recruitment domain 4; AIM2, absent in melanoma2; ASC, adaptor protein apoptosis associated speck like proteins; LPS, lipopolysaccharide; DFNA5, deafness autosomal dominant 5; GSDME, gasdermin E; ROS, reactive oxygen species; GZMB, Granzyme B; LDH, lactate dehydrogenase; NSCLC, nonsmall-cell lung cancer; siRNA, small interfering RNA; MIP, macrophage inflammatory protein; IP-10, interferon-inducible protein-10; TTM, Trillium tschonoskii Maxim; PPVI, Polyphyllin VI; PG, Emodin 80O-β-glucopyranoside; miRNA, microRNA; DHA, docosahexaenoic acid; PD-L1, programmed death ligand 1; PD-1, programmed cell death protein 1; nPD-L1, nuclear programmed death ligand 1; GSDMC, gasdermin C; HCC, hepatocellular carcinoma; E2, 17β-estradiol; AIF, Alpinumisoflavone; 5-FU, 5-Fluorouracil; ERK, extra Cellular signal-regulated kinase; STAT3, Signal Transducer and Activator of Transcription 3; PI3K, phosphatidylinositol 3 kinase; NK cell, natural killer cell; TILs, tumor infiltrating lymphocytes; PFN, perforin; CAR-T, chimeric antigen receptor T cell; CRS, cytokine release syndrome; CTL, Cytotoxic T Lymphocyte.
